# Prolonged membrane potential depolarization in cingulate pyramidal cells after digit amputation in adult rats

**DOI:** 10.1186/1744-8069-1-23

**Published:** 2005-08-19

**Authors:** MF Wu, ZP Pang, M Zhuo, ZC Xu

**Affiliations:** 1 Department of Anatomy & Cell Biology, Indiana University School of Medicine, Indianapolis, IN, 46202, USA; 2 University of Texas Southwestern Medical Center, Dallas, TX, 75390, USA; 3 Department of Physiology, Faculty of Medicine, University of Toronto, University of Toronto Centre for the Study of Pain, Toronto, M5S 1A8, Canada

## Abstract

The anterior cingulate cortex (ACC) plays an important role in higher brain functions including learning, memory, and persistent pain. Long-term potentiation of excitatory synaptic transmission has been observed in the ACC after digit amputation, which might contribute to plastic changes associated with the phantom pain. Here we report a long-lasting membrane potential depolarization in ACC neurons of adult rats after digit amputation *in vivo*. Shortly after digit amputation of the hind paw, the membrane potential of intracellularly recorded ACC neurons quickly depolarized from ~-70 mV to ~-15 mV and then slowly repolarized. The duration of this amputation-induced depolarization was about 40 min. Intracellular staining revealed that these neurons were pyramidal neurons in the ACC. The depolarization is activity-dependent, since peripheral application of lidocaine significantly reduced it. Furthermore, the depolarization was significantly reduced by a NMDA receptor antagonist MK-801. Our results provide direct *in vivo *electrophysiological evidence that ACC pyramidal cells undergo rapid and prolonged depolarization after digit amputation, and the amputation-induced depolarization in ACC neurons might be associated with the synaptic mechanisms for phantom pain.

## 

The anterior cingulate cortex (ACC) is involved in sensory signal processing such as integration of general affect, cognition, and pain unpleasantness [1-6]. Neurons in the ACC respond to peripheral nociceptive stimulation [7-10]. It has been shown that digit amputation causes a long-lasting potentiation of the ACC responses to peripheral electrical stimulation, which might contribute to the phantom limb pain [10,11]. Phantom limb pain is experienced in a limb that is no longer present. About 50–80% amputees suffer from phantom limb pain [12,13]. Recent studies have indicated that cortical reorganization occurs following digit or limb amputation [14,15], which correlates with phantom limb pain or phantom limb sensation in most amputees [12,13]. However, less information is available about the possible early changes within the ACC after amputation. Recent studies using *in vitro *brain slice preparations showed that digit amputation in rats caused the loss of long-term depression (LTD) in the ACC [7]. Here we employed intracellular recordings from anesthetized adult rats and reported a prolonged membrane depolarization in ACC neurons after digit amputation of the hind paw.

Male adult Wistar rats (200–350 g) were anesthetized with 1–2% halothane. The core body temperature was maintained at 37°C with a heating pad. In vivo intracellular recording was performed as previously described [16,17]. Briefly, the animals were placed in a stereotaxic frame. A craniotomy was performed in the region above the left ACC (AP: 1.0–3.7 mm, RL: 0.5–1.0 mm, H: 1.3–3.0 mm, Bregma) [18]. The intracellular recording electrodes had a tip resistance of 40–70 M_ as filled with a solution of 4% neurobiotin (Vector, CA, USA) in 2 M potassium acetate. The microelectrode was advanced slowly into the left ACC to impale neurons. After impalement, the neurons with a stable membrane potential of -60 mV or greater and action potential amplitudes of at least 60 mV were selected for further study. After a baseline recording, the 3^rd ^digit of the hind paw was amputated with deep anesthesia (2–3% halothane). In some experiments, extracellular recording was simultaneously conducted in the ACC with intracellular recording. The extracellular recording electrodes had a tip resistance of ~4 M_ as filled with a solution of 2 M sodium chloride. One femoral vein was cannulated in some animals for drug delivery. Data were digitized and stored with a Macintosh computer using the data acquisition program Axodata (Axon Instruments, CA, USA). Values were presented as mean ± S.E.M. Analysis of variance (ANOVA) followed by post-hoc Fisher's test was used for statistical analysis. Changes were considered significant if P < 0.05.

After each successful recording, neurobiotin was iontophoresed into the cell by passing depolarizing current pulses (2 Hz, 300 ms, 1.0–1.5 nA) for 10–20 min. At the end of the experiment, the animal was deeply anesthetized and perfused transcardially with 0.01 M phosphate-buffered saline followed by 4% paraformaldehyde. The brain was removed and stored in fixative overnight. Coronal sections were cut at 80 μm thickness using a Vibratome (Ted Pella, CA, USA) and incubated in 0.1% horseradish peroxidase-conjugated avidin-D (Vector, CA, USA) in 0.01 M potassium phosphate-buffered saline (KPBS, pH 7.4) with 0.5% Triton X-100 for 6–8 h at room temperature. After detection of peroxidase activity with 3,3'-diaminobenzidine, the sections were examined in KPBS. The sections containing labeled neurons were mounted on gelatin-coated slides and processed for light microscopy.

A total of 77 rats were used in the present study. Among these animals, intracellular and extracellular recording were performed simultaneously in 38 rats. In order to compare electrophysiological responses in ACC neurons before and after digit amputation, only one neuron was recorded from each animal. All intracellularly stained neurons were successfully recovered, among which 36 were pyramidal neurons in ACC. Two neurons were located in other regions of the brain and were excluded from the present study. An example of intracellularly stained ACC pyramidal neurons is presented in Fig 1. Two neurons were excluded from the present study because they were identified as neurons in other regions of the cortex. Because maintaining a prolonged intracellular recording is extremely difficult, extracellular recording alone was performed in the rest of animals (n = 39). The DC potential response recorded by extracellular recording exhibited the same profiles as that of intracellular recording, so the data from intracellular recordings and extracellular recordings were pooled together.

**Figure 1:**
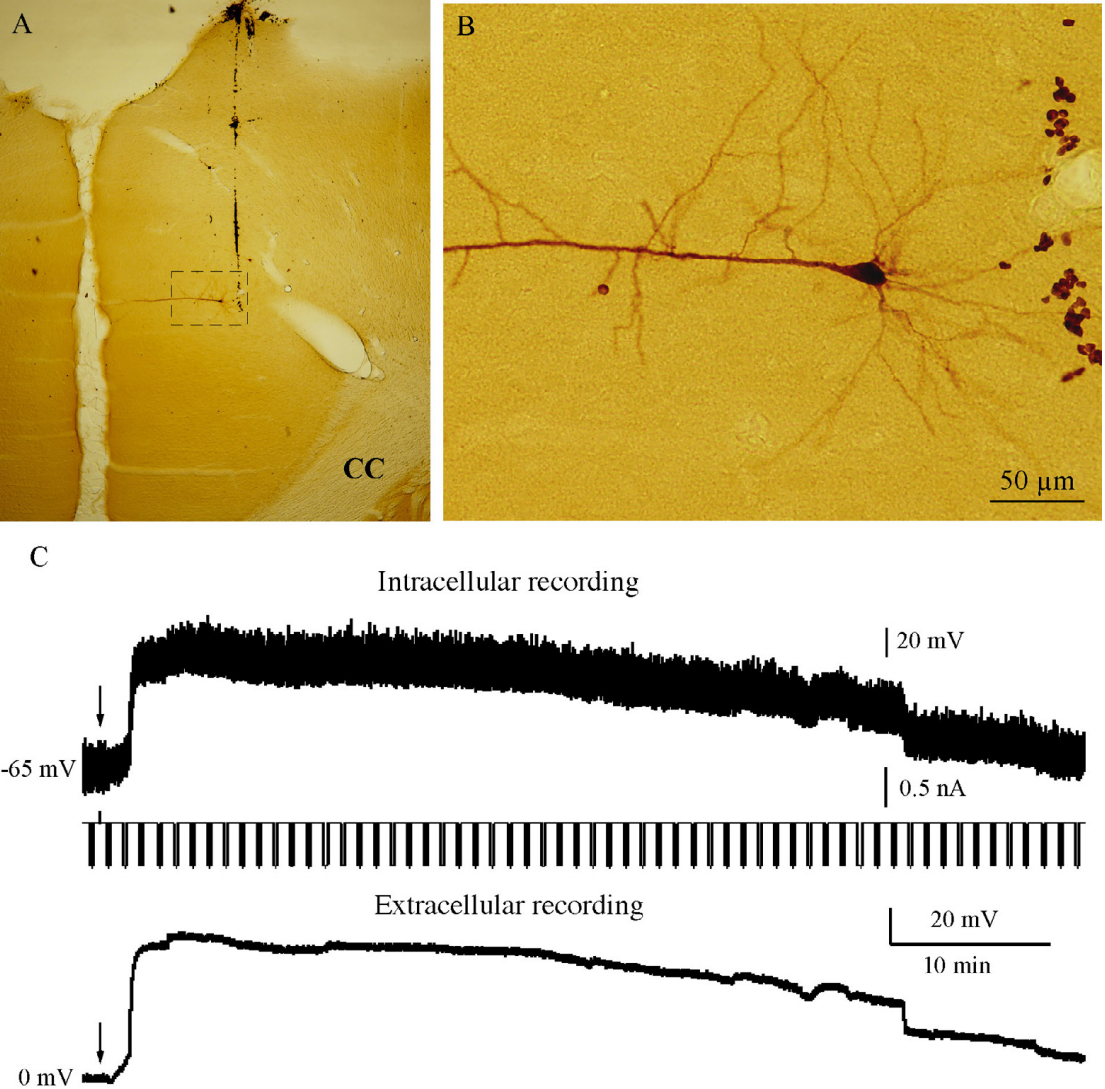
***Prolonged membrane potential change of ACC neurons after digit amputation***. A. Light photomicrograph of a pyramidal neuron (square) in the ACC intracellularly stained with neurobiotin after recording. B. High magnification of the labeled pyramidal neuron in Figure A. C. Representative recordings showing the response of ACC neuron to the contralateral third digit amputation. The upper panel is the membrane potential intracellularly recorded from a pyramidal neuron, the middle panel is the intracellularly applied hyperpolarizing current pulses (1 Hz, -0.5 nA, 200 ms), the lower panel is the simultaneous extracellular recording of DC potential in the ACC region. The arrows indicate the time of digit amputation. Approximately 2 min after amputation, the baseline membrane potentials from both recording quickly depolarize for approximately 50 mV and gradually returned to the control level in about 55 min.

After digit amputation, a dramatic membrane potential depolarization occurred in approximately 70% of the neurons (or animals) recorded in ACC (52/75). The depolarization occurred shortly after amputation with a latency of 0.52 ± 0.08 min (n = 20) and quickly reached the maximal amplitude in approximately 2 minutes. The amplitude of this amputation-induced depolarization was 53.5 ± 4.0 mV (n = 24) with the membrane potential shifted from -71.9 ± 1.2 mV to -15.1 ± 5.3 mV from intracellular recordings (n = 15) and from 0 mV to 50.1 ± 7.0 mV from extracellular recordings (n = 9). The membrane potential slowly repolarized and returned to the control levels. The duration of this depolarization was 43.0 ± 4.5 min (n = 12). During intracellular recording, hyperpolarizing current pulses were delivered to monitor the membrane input resistance, which was indicated by the amplitude of membrane potential deflection. No obvious changes in membrane input resistance were detected during the amputation-induced depolarization. Representative traces of simultaneous intracellular and extracellular recording of amputation-induced depolarization is shown in Fig 1C. The onset, time course, and the amplitude of these two traces were almost identical.

To elucidate the possible mechanisms underlying the amputation-induced depolarization, lidocaine (2%, 1 ml, s.c.) was locally injected into the right hind paw 5 min before the digit amputation. In animals with lidocaine injection, the amplitude of depolarization significantly reduced to 34.0 ± 6.5 mV (n = 10, *P *< 0.01) and the duration dramatically shortened to 13.2 ± 2.8 ms (n = 8, *P *< 0.01). However, the latency of the onset of depolarization in lidocaine treated animals remained about the same as of control ones (Table 1). These results suggest that the amputation-induced depolarization is associated with the nociceptive stimulation.

**Table 1:** Effects of lidocaine and glutamate receptor antagonists on membrane depolarization

	Control	Lidocaine	MK-801
Latency	0.52 ± 0.08 (n = 20)	0.48 ± 0.08 (n = 10)	1.53 ± 0.15* (n = 10)
Amplitude (mV)	53.5 ± 4.0 (n = 24)	34.0 ± 6.5* (n = 10)	28.5 ± 2.1* (n = 10)
Duration	43.0 ± 4.5 (n = 12)	13.2 ± 2.8* (n = 8)	23.3 ± 6.0* (n = 8)

*, *P *< 0.01 compared with control

It has been shown that the glutamatergic activation is involved in the pain signal processing in ACC [1,19,20]. To reveal the involvement of glutamate receptors in amputation-induced depolarization, NMDA receptor or non-NMDA receptor antagonists were applied intravenously 5 min before the digit amputation. In animals received NMDA receptor blocker MK801 (1 mg/kg), the amplitude of depolarization significantly decreased to 28.5 ± 2.1 mV (n = 10, *P *< 0.01) and the duration reduced to 23.3 ± 6.0 min (n = 8, *P *< 0.01). In contrast, the latency to the onset of depolarization was significantly prolonged to 1.53 ± 0.15 min (n = 10, *P *< 0.01) (Table 1). These data indicate that the amputation-induced depolarization is mediated by NMDA receptors.

Accumulated evidence indicates that the ACC is involved in pain processing. Surgical ablation or electrolytic lesion of the ACC reduces pain related unpleasantness [2]. Activation of opioid receptors in the ACC is accompanied by a reduction in pain [4]. A number of functional imaging studies have shown consistently the ACC activation during application of noxious stimuli to various parts of the body [5,21,22]. Recent reports demonstrate that the digit amputation causes the enhancement of sensory responses in the ACC and the loss of LTD in in vitro ACC slices for at least 2 weeks [7,10]. Interestingly, in the present study, we observed a dramatic membrane depolarization in ACC neurons after digit amputation. Dramatic membrane depolarization has been observed during cortical spreading depression or cerebral ischemia [23,24]. Cortical spreading depression could be triggered by chemical, electrical or mechanical stimulation [25] while anoxic or ischemic depolarization is mainly caused by the failure of Na^+^-K^+ ^pump due to energy depletion [26]. The depolarization in ACC neurons after amputation is most likely elicited by nociceptive stimulation as local anesthesia could significantly reduce the size of the depolarization. However, local application of lidocaine couldn't completely block the amputation-induced depolarization. Besides the possible insufficient dosage, it is possible that subcutaneous injection of lidocaine fails to block the sensory afferent fibers from the deep structures such as bones and joints.

Glutamate is a major excitatory neurotransmitter in the central nervous system including the ACC [7]. Growing evidence has recently shown that NMDA receptors are involved in synaptic plasticity related to injury [2,27]. NMDA receptors are highly expressed in the ACC [2]. It has been demonstrated that NMDA receptors play important roles in mediation of pain signal processing in the ACC [7,28]. Data from the present study suggest that activation of NMDA receptor is mainly responsible to the depolarization elicited by digit amputation. Coincided with our observation, NMDA receptor antagonist MK801 blocks the cortical spreading depression [29]. It is conceivable that membrane potential depolarization induced by amputation results in the enhancement of Ca^2+ ^influx through voltage-dependent Ca^2+ ^channels and NMDA receptors, which might initiate the intracellular events leading to the alteration of synaptic transmission. A recent study showed that calcium-stimulated adenylyl cyclase including AC1 and AC8 may play critical roles in inducing long-term potentiation in the ACC neurons [30], behavioral nociceptive responses to inflammation and tissue injury as well as allodynia related to nerve injury [31]. The prolonged postsynaptic membrane depolarization in the ACC pyramidal cells provides a novel induction mechanism for triggering long-term plastic changes within the ACC such as LTP and LTD. These rapid onset and prolonged changes in excitatory synaptic transmission activate a series of signaling pathways including stimulating new protein synthesis that may play important roles in amputation related cortical reorganization as reported in monkeys and humans.

## List of abbreviations

NMDA: *N*-methyl-D-aspartate

AMPA: α-amino-3-hydroxy-5-methyl-isoxozole propionic acid

KA: kainate MK801: (+)-5-methyl-10, 11-dihydro-5H-dibenzo [*a*, *d*] cyclohepten-5, 10-imine hydrogen maleate
